# Bioassay-Guided Isolation of Anti-Inflammatory Components from the Bulbs of *Lilium brownii* var. *viridulum* and Identifying the Underlying Mechanism through Acting on the NF-κB/MAPKs Pathway

**DOI:** 10.3390/molecules22040506

**Published:** 2017-03-23

**Authors:** Ting Ma, Zhen Wang, Yang-Mei Zhang, Jian-Guang Luo, Ling-Yi Kong

**Affiliations:** State Key Laboratory of Natural Medicines, Department of Natural Medicinal Chemistry, China Pharmaceutical University, 24 Tong Jia Xiang, Nanjing 210009, China; maxueting0842910@163.com (T.M.); wangzhen_199202@163.com (Z.W.); 13812775276@163.com (Y.-M.Z.)

**Keywords:** *Lilium brownii* var. *viridulum*, inflammation, nitric oxide, RAW264.7 cells, mitogen-activated protein kinases, nuclear factor-κB

## Abstract

The bulbs of *Lilium brownii* var. *viridulum* (LB) are commonly used as both traditional Chinese medicines and popular functional food for many centuries in China. Previous studies reported that the extract of lily bulbs exhibited anti-inflammatory activity both in vivo and in vitro, but its active components and associated molecular mechanisms remain elusive. In the present study, using bioassay-guided isolation method, two phenylpropenoid acylglycerols, 1-*O*-feruloyl-2-*O*-*p*-coumaroylglycerol (**1**) and 1,3-*O*-diferuloylglycerol (**2**), were obtained and identified from the chloroform fraction of LB. Both compounds **1** and **2** significantly decreased the production of nitrite oxide (NO) in lipopolysaccharide (LPS)-stimulated mouse macrophage RAW264.7 cells in a dose-dependent manner with half maximal inhibitory concentration (IC_50_) values of 9.12 ± 0.72 μM and 12.01 ± 1.07 μM, respectively. They also inhibited the production of prostaglandin E2 (PGE2) and several other pro-inflammatory cytokines, such as interleukin-1β (IL-1β), interleukin-6 (IL-6), and tumor necrosis factor-α (TNF-α). Furthermore, compounds **1** and **2** downregulated the protein levels of inducible nitric oxide synthase (iNOS) and cyclooxygenase-2 (COX-2). They also inhibited the nuclear translocation of nuclear factor-κB (NF-κB) p65 subunit and suppressed mitogen-activated protein kinases (MAPKs) pathway. Taken these data together, compounds **1** and **2** exhibited anti-inflammatory activities through acting on the NF-κB and MAPKs pathway. This research provides the first evidence on the major bioactive constituents and related molecular mechanisms of LB as an anti-inflammatory agent. Our findings also advanced the understanding of LB as a traditional herbal medicine for the prevention and treatment of inflammation.

## 1. Introduction

*Lilium brownii* var. *viridulum* Baker (Lily), belonging to the family Liliaceae, is widely distributed and planted in northern and eastern Asia. Its bulbs (LB), commonly known as “Bai-he” and officially listed in Chinese Pharmacopoeia, have long been used as traditional Chinese medicines for treatment of chronic gastritis, pertussis, pneumonia, bronchitis and cough. Moreover, lily bulbs are an important part of the Asian diet and are regularly consumed as functional food due to their primary abilities of nourishing yin, moistening lung and clearing heartburn according to the traditional theories of Chinese medicine [1,2,3]. Moreover, recent pharmacological studies suggested that the extract of lily bulbs possesses anti-inflammatory activity in both cells and cigarette smoke-exposed mouse models [4,5]. However, the bioactive compounds responsible for its anti-inflammatory effect and the relevant underlying mechanisms have not been disclosed yet.

Inflammation is a physiological defense reaction of living organisms against harmful stimuli, such as damaged cells, pathogens, or irritants. It leads to the classical syndromes of heat, redness, swelling and hyperalgesia. Furthermore, deregulated activation of inflammation has been considered to be one of the principal causes of inflammatory related diseases such as rheumatoid arthritis, Alzheimer’s disease, diabetes and even cancers [6,7,8,9]. Thus, maintaining the normal occurrence and development of inflammation is of significant importance. As we all know, inflammatory response and tissue damage are usually induced by inflammatory cytokines (tumor necrosis factor-α (TNF-α), interleukin-6 (IL-6) and interleukin-1β (IL-1β)) and related inflammatory mediators, including nitric oxide (NO) and prostaglandin E2 (PGE2) produced by inducible nitric oxide synthase (iNOS) and cyclooxygenase (COX-2), respectively [10,11,12]. Therefore, suppressing the release of these inflammatory cytokines and mediators may control or relieve tissue injury during the inflammatory process.

Several studies have reported that macrophages play a central role in host defenses against noxious substances [13,14]. Macrophage activation by lipopolysaccharide (LPS) increases the production of pro-inflammatory cytokines and the expression of iNOS and COX-2, resulting in enhanced inflammatory response and injury to cells or tissues [15,16]. Therefore, LPS-stimulated macrophages could be used as a model to study the occurrence of inflammation and mechanisms of potential anti-inflammatory action. Inhibition of pro-inflammatory cytokines and mediators released from LPS-induced macrophages is always used to evaluate the activity of anti-inflammatory agents. Additionally, previous studies have found that the mitogen-activated protein kinase (MAPK) and the nuclear factor κB (NF-κB) pathways are activated in LPS-stimulated macrophages, modulating the expression of iNOS and COX-2 and assisting inflammatory response [17,18,19]. Thus, targeting the NF-κB and MAPK pathways might be potential therapeutic strategies to control some inflammatory diseases.

In this study, we made use of the bioassay-guided approach to investigate the potential anti-inflammatory constituents of LB and macrophage RAW264.7 cell line to explore the molecular mechanisms responsible for its anti-inflammatory activity. Two phenylpropenoid acylglycerols, 1-*O*-feruloyl-2-*O*-*p*-coumaroylglycerol (**1**) and 1,3-*O*-diferuloylglycerol (**2**) were found to exhibit significant inhibitory effects on LPS-induced NO production in RAW264.7 cells. Herein, we report the bioassay-guided isolation and NO inhibitory screening of compounds **1** and **2** as well as their underlying anti-inflammation mechanism on RAW264.7 cells. These results also expanded the application of LB as bioactive ingredients in functional foods and traditional herbal medicines for the prevention and treatment of inflammation.

## 2. Results

### 2.1. Phytochemical and Bioactive Screening of the Crude Extracts and Fractions of LB

**Figure 1:**
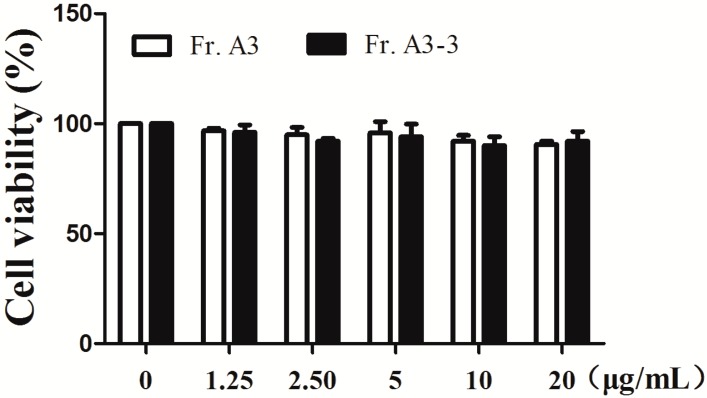
Effects of Fr. A3 and Fr. A3-3 on cell viability. Cells were treated with Fr. A3 and Fr. A3-3 for 1 h and administered with lipopolysaccharide (LPS, 1 μg/mL) for 24 h, then cell viability was measured by 3-[4,5-dimethylthiazol-2-yl]-2,5-diphenyl tetrazolium bromide (MTT) assay.

**Figure 2:**
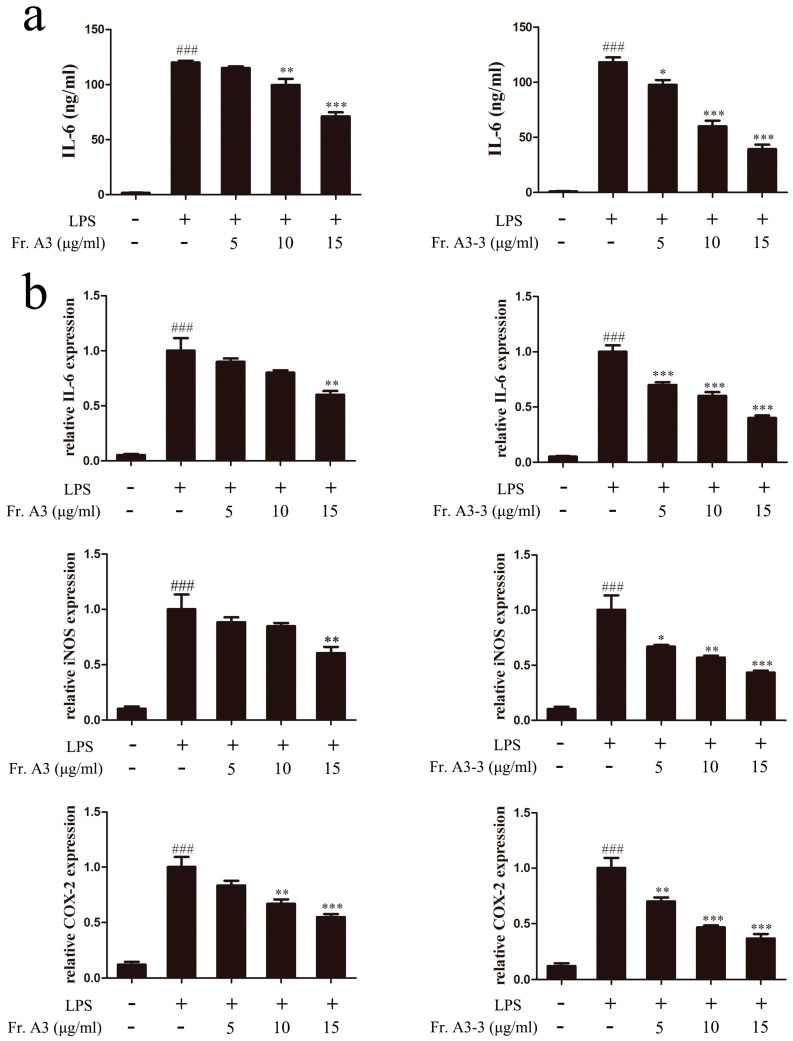
Effects of Fr. A3 and Fr. A3-3 on LPS-induced interleukin-6 (IL-6) release and the gene expression levels of IL-6, inducible nitric oxide synthase (iNOS) and cyclooxygenase-2 (COX-2). (**a**) Cells were pretreated with Fr. A3 and Fr. A3-3 in the presence of LPS (1 μg/mL) for 18 h. IL-6 level was measured by enzyme linked immunosorbent assay (ELISA) assay; (**b**) Gene levels of IL-6, iNOS and COX-2 expression were determined by quantitative real-time polymerase chain reaction (qRT-PCR). ^###^ *p* < 0.001, compared to the control group; * *p* < 0.05, ** *p* < 0.01, *** *p* < 0.001, compared to the LPS-treated group.

For the crude MeOH extract of LB (LBM) and partitioned extracts of LBM, we conducted anti-inflammatory screening and results were shown in Table 1. The crude MeOH extract showed moderate anti-inflammatory activity with half maximal inhibitory concentration (IC_50_) value of 71.52 ± 4.52 μg/mL for inhibiting NO production. Further partitioned extracts of LBM by different kinds of organic solvents (CHCl_3_, EtOAc and *n*-BuOH) showed significant improvement on the anti-inflammatory activity, of which Fr. A (CHCl_3_) and Fr. C (*n*-BuOH) had the IC_50_ value of 32.70 ± 1.56 and 40.70 ± 0.92 μg/mL, respectively. However, Fr. C exhibited cytotoxicity in RAW264.7 cells at the same time. Therefore, Fr. A was chosen for further research and fractionated into five sub-fractions using silica gel column chromatography, and then all the sub-fractions were tested for their abilities against NO production. The results in Table 1 suggested that Fr. A3 had the lowest IC_50_ value of 11.49 ± 0.69 μg/mL compared with Fr. A and other sub-fractions. Furthermore, Fr. A3 was subjected to repeat column chromatography to give seven sub-fractions, of which Fr. A3-3 possessed the strongest activity against NO production. At the same time, we measured the cytotoxicity effect of Fr. A3 and Fr. A3-3 by 3-[4,5-dimethylthiazol-2-yl]-2,5-diphenyl tetrazolium bromide (MTT) assay to exclude their cytotoxic effect on RAW264.7 cells. The results suggest that Fr. A3 and Fr. A3-3 had no obvious cytotoxicity at doses of 1.25–20 μg/mL (Figure 1). Thus, in the subsequent experiments, the doses of 5–15 μg/mL were chosen to conduct further research. To confirm the anti-inflammatory effect of Fr. A3 and its sub-fraction Fr. A3-3, the level of pro-inflammatory cytokine IL-6 was evaluated and results demonstrated that 15 μg/mL of Fr. A3 and Fr. A3-3 inhibited IL-6 release by 41.4% and 68.1%, respectively, compared to LPS induced group as illustrated in Figure 2a. Additionally, Figure 2b indicated that the gene levels of IL-6, iNOS and COX-2 were also significantly decreased by Fr. A3 and Fr. A3-3 in LPS-stimulated macrophage cells. From the results, we conclude that the Fr. A3 and its sub-fraction Fr. A3-3 were main bioactive ingredients for anti-inflammatory activity of LB.

### 2.2. Identification of Active Compounds and Evaluation of Their Cytotoxicities in RAW264.7 Macrophages

**Figure 3:**
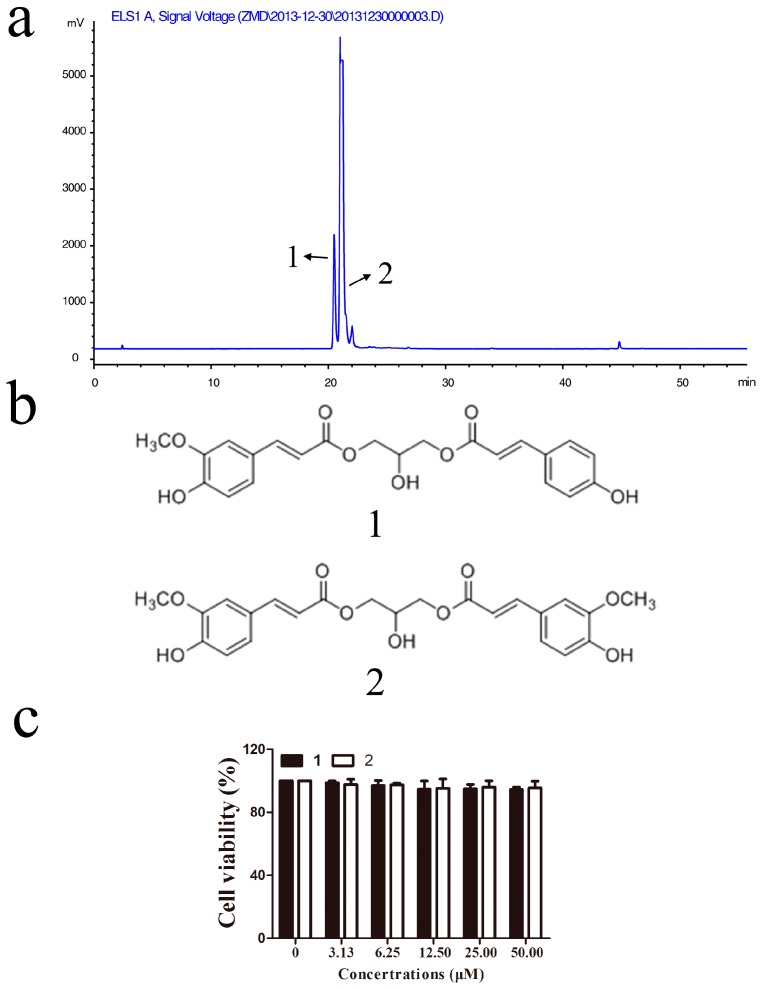
Identification of compounds and their cytotoxicities in RAW264.7 macrophages: (**a**) High-performance liquid chromatography (HPLC) chromatogram of Fr. A3-3; (**b**) chemical structures of compounds **1** and **2**; and (**c**) effects of **1** and **2** on cell viability. Cells were treated with compounds **1** or **2** for 1 h and administered with LPS (1 μg/mL) for 24 h, then cell viability was measured by MTT assay.

To find the active compounds, the Fr. A3-3 fraction was analyzed by high-performance liquid chromatography (HPLC) to find major peaks (Figure 3a) and further purified for the purpose of identifying the active chemical ingredient. Finally, this fraction afforded two known phenylpropenoid acylglycerols, 1-*O*-feruloyl-3-*O*-*p*-coumaroylglycerol (**1**) and 1,3-*O*-diferuloylglycerol (**2**) (Figure 3b). These two compounds were both previously obtained from the bulbs of *L. auratum* [20] and *L. lancifolium* [21], and compound **1** was also isolated from the aerial parts of *Tillandsia streptocarpa* [22]. Data of each compound were given in the Materials and Methods section.

To examine possible cytotoxicity of isolated compounds in RAW264.7 macrophages cells, MTT assay was conducted to determine the cytotoxicity effect. As reflected in Figure 3c, RAW264.7 cell viability was not significantly changed by compounds **1** or **2** treatment at concentrations of 3–50 μM within 24 h incubation. Thus, compounds **1** or **2** had no cytotoxicities against RAW 264.7 cells in this study.

### 2.3. Anti-Inflammatory Activity Study of Compounds ***1*** and ***2***

**Figure 4:**
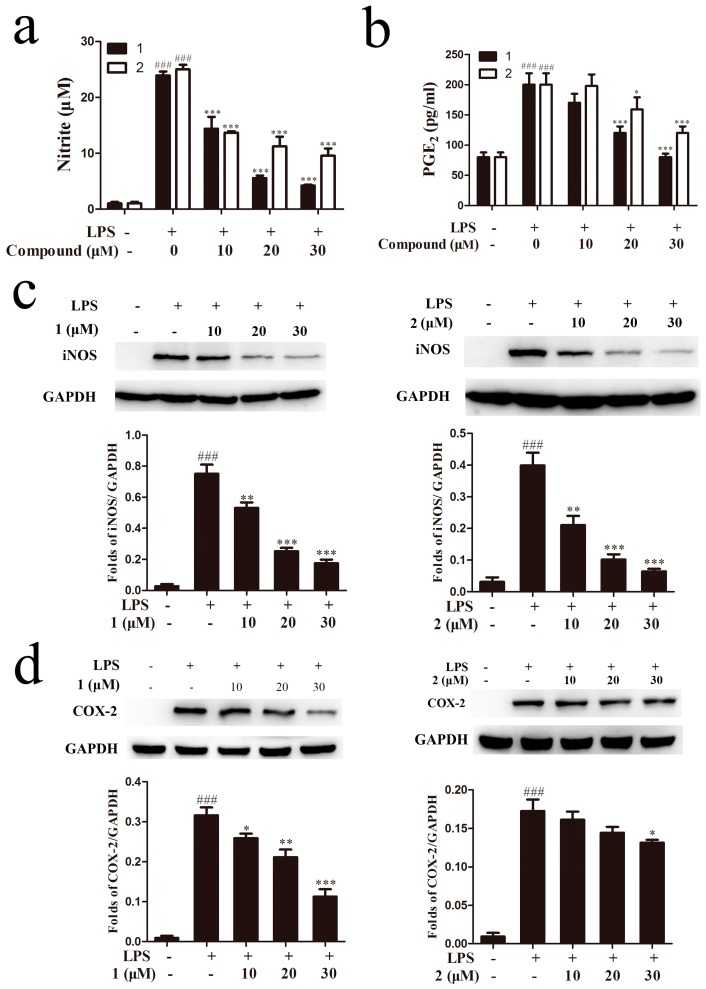
Anti-inflammatory activity study of compounds **1** and **2**. Cells were treated with compounds **1** or **2** for 1 h and then co-incubated with 1 μg/mL LPS for 18 h. The NO production was determined by Griess assay (**a**) and the release of prostaglandin E2 (PGE2) was measured by ELISA assay (**b**); RAW264.7 cells were pretreated with compounds **1** or **2** and then incubated with LPS (1 μg/mL) for 18 h. Western blot assay was used to detect iNOS (**c**) and COX-2 protein expression (**d**). ^###^ *p* < 0.001, compared to the control group; * *p* < 0.05, ** *p* < 0.01, *** *p* < 0.001, compared to the LPS-treated group. GAPDH: glyceraldehyde-3-phosphate dehydrogenase.

Nitric oxide is an important signaling molecule that has critical functions in host immune defense, vascular regulation, neuronal signal transduction, and other systems [23]. High NO levels are generated in response to inflammatory stimuli and NO production is tightly involved in the occurrence of inflammation. Therefore, the anti-inflammation effects of drugs can be evaluated by monitoring the NO level. To investigate the inhibitory effects of isolated compounds on LPS-induced NO production, cells were treated with or without compounds **1** or **2** (10, 20, 30 μM), followed by stimulation with LPS (1 μg/mL) for 18 h. As shown in Figure 4a, compared with control cells, stimulation of cells with LPS led to significant increase of the nitrite concentration in conditioned medium. Compounds **1** and **2** obviously decreased NO production in a concentration dependent manner with IC_50_ values of 9.12 ± 0.72 μM and 12.01 ± 1.07 μM, respectively. As another mediator of inflammation, PGE2 is produced by the metabolism of arachidonic acid via COX enzymes. Our results demonstrated that compounds **1** and **2** at 30 μM caused a decrease of PGE2 release about 96% and 67%, respectively, in activated RAW264.7 cells (Figure 4b). Considering that NO was the product of iNOS, we further investigated the effect of compounds **1** and **2** on LPS-induced expression of iNOS in RAW264.7 cells. LPS-treatment obviously increased iNOS protein expression compared to the non-treated groups, while such a high iNOS expression was significantly decreased by compounds **1** and **2** at the concentrations of 10–30 μM, especially at the dose of 30 μM, the protein level of iNOS was decreased to almost the same level as the normal group (Figure 4c). The results suggested that the two compounds inhibited NO production by suppressing iNOS protein level. COX-2 expression was induced by external stimuli in mammalian tissues and was the general target of anti-inflammatory treatment [24]. To determine whether tested compounds also affected COX-2 protein expression, western blot analysis was performed to assess the level of COX-2 protein. As indicated in Figure 4d, we found that 30 μM compound **1** and **2** inhibited the protein expressions of COX-2 (*p* < 0.001 and *p* < 0.05, respectively). Taking these data together, compounds **1** and **2** exhibited powerful anti-inflammatory effect in LPS-stimulated RAW264.7 cells through inhibiting NO production and iNOS and COX-2 protein expressions.

### 2.4. Inhibitory Effects of Compounds ***1*** and ***2*** on TNF-α, IL-6 and IL-1β Expressions

**Figure 5:**
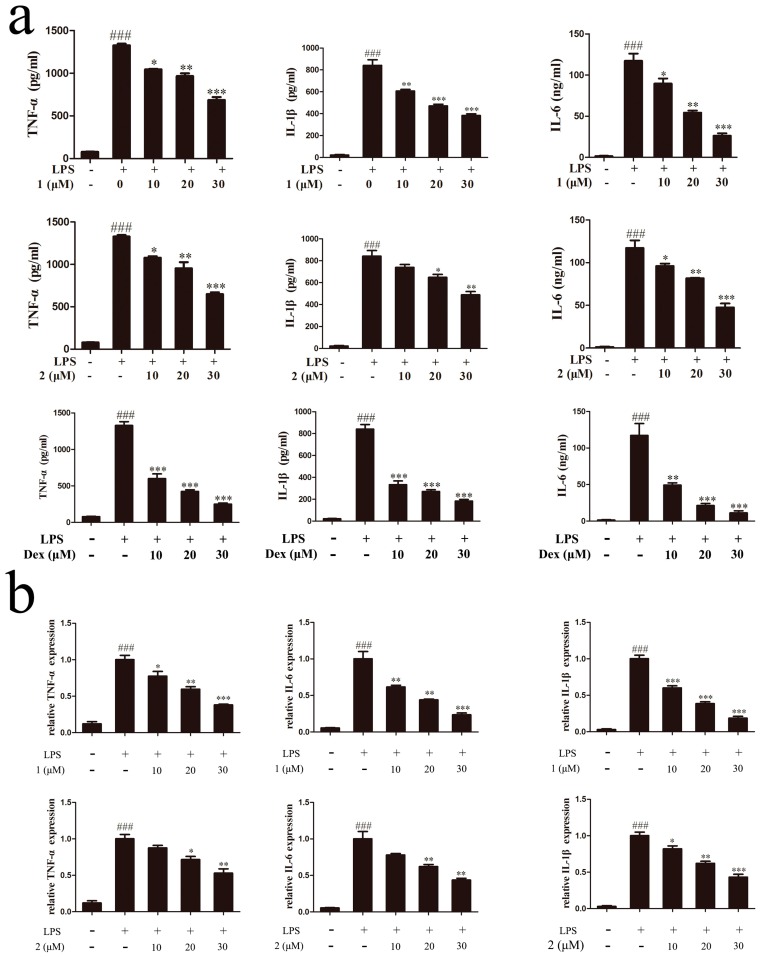
Inhibitory effect of compounds **1** and **2** on TNF-α, IL-6 and IL-1β expressions. (**a**) Cells were treated with compound **1** or **2** or dexamethasone for 1 h and then stimulated with 1 μg/mL LPS for 18 h. Production of TNF-α, IL-6, and IL-1β was examined by ELISA assay. (**b**) TNF-α, IL-6, and IL-1β gene expression levels were measured by qRT-PCR. ^###^ *p* < 0.001, compared to the control group; * *p* < 0.05, ** *p* < 0.01, *** *p* < 0.001, compared to the LPS-treated group.

Inflammatory cytokines such as TNF-α, IL-6, and IL-1β play an important role in the process of inflammation [25,26]. To further ascertain the anti-inflammatory effect of compounds **1** and **2** in LPS-induced RAW264.7 cells, specific enzyme linked immunosorbent assay (ELISA) kits were used to evaluate the release of pro-inflammatory cytokines, including TNF-α, IL-1β and IL-6. In our experiment, dexamethasone with an IC_50_ value of 1.73 μM for inhibiting NO production was chosen as the positive control drug. As demonstrated in Figure 5a, production of TNF-α, IL-1β and IL-6 was clearly increased in LPS-stimulated RAW264.7 cells compared with non-treated counterparts. As was expected, the levels of all three cytokines were markedly reduced by pretreatment with different concentrations of compounds **1** or **2** as well as dexamethasone. The inhibition effect of TNF-α, IL-6, and IL-1β was also summarized in Table 2. From the data, we got that the pro-inflammatory cytokines inhibition effect of compounds **1** and **2** was weaker than the positive drug dexamethasone at the same dose, but they also exhibited the similar effects as dexamethasone on inhibiting the release of TNF-α, IL-6, and IL-1β. Furthermore, the effect of compounds **1** and **2** on the mRNA levels of TNF-α, IL-6, and IL-1β were also determined by quantitative real-time polymerase chain reaction (qRT-PCR). Results suggested that LPS stimulation significantly increased TNF-α, IL-6, and IL-1β mRNA levels, while pretreatment of the two compounds dose-dependently decreased TNF-α, IL-6, and IL-1β gene levels (Figure 5b). These findings indicated that both of the compounds inhibited excessive pro-inflammatory cytokines release and gene expressions of TNF-α, IL-6, and IL-1β in LPS-activated RAW264.7 cells.

### 2.5. Inhibition of MAPKs and NF-κB Signaling Pathways Accounts for the Anti-Inflammatory Effect of Compounds ***1*** and ***2***

**Figure 6:**
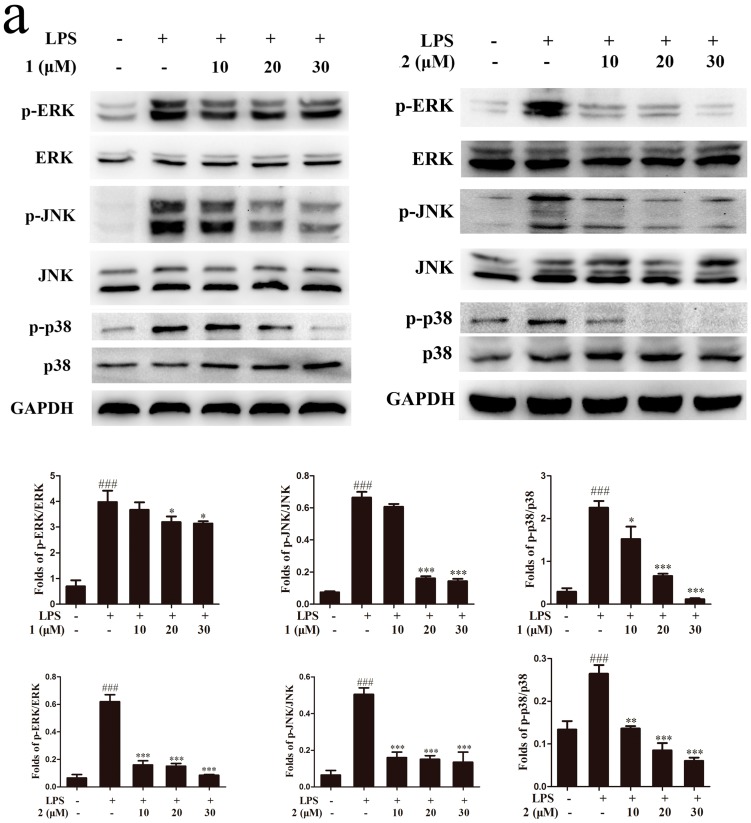

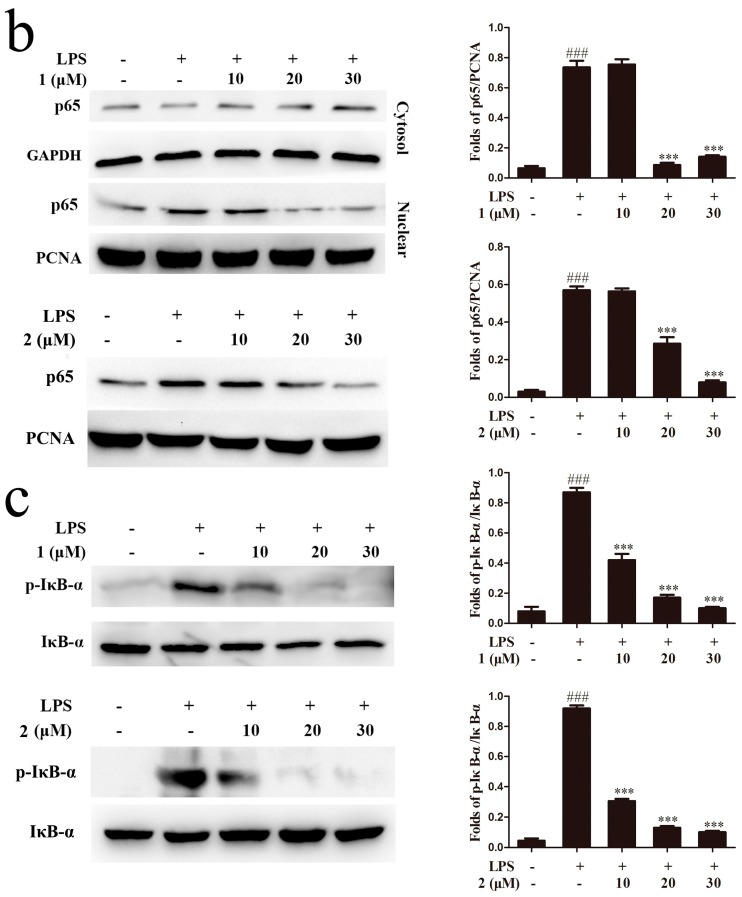
The anti-inflammatory mechanism study of compounds **1** and **2**. RAW264.7 cells (10^6^ cells/mL) were pretreated with different concentrations of **1** and **2** for 1 h and then incubated with LPS (1 μg/mL) for 18 h. (**a**) Protein levels of phosphorylated extracellular signal-regulated kinase (p-ERK), extracellular signal-regulated kinase (ERK), phosphorylated c-jun NH_2_-terminal kinase (p-JNK), c-jun NH_2_-terminal kinase (JNK), p38 mitogen activated protein kinase (p38) and phosphorylated p38 mitogen activated protein kinase (p-p38) were analyzed by using Western blotting; (**b**) Effects of **1** and **2** on nuclear translocation of NF-κB p65 were analyzed by using Western blotting; (**c**) Phosphorylation of I kappa B alpha (IκB-α) was analyzed by Western blotting. ^###^ *p* < 0.001, compared to the control group; * *p* < 0.05, ** *p* < 0.01, *** *p* < 0.001, compared to the LPS-treated group. PCNA: proliferating cell nuclear antigen.

The MAPKs pathway is one of the most extensively investigated signal transduction pathways related to inflammation process. Previous studies have reported that MAPKs pathway plays an important role during the release of pro-inflammatory cytokines and inflammatory mediators in LPS-induced RAW264.7 cells [27,28]. Therefore, we examined the effects of compounds **1** and **2** on the phosphorylation levels of p38 MAPK (p38), extracellular signal-regulated kinase (ERK) and c-jun NH_2_-terminal kinase (JNK) in the presence of LPS stimulation. As reflected in Figure 6a, LPS treatment increased phosphorylation level of MAPKs in RAW264.7 cells, whereas pretreatment with compound **1** or **2** significantly attenuated the phosphorylation levels of p38, JNK and ERK, with no obvious changes in the total protein expressions. These results indicated that compounds **1** and **2** inhibited inflammatory mediator expression and pro-inflammatory cytokines production, at least in part, by suppressing the MAPKs signaling pathway. As another important transcription factor, NF-κB was also reported to regulate the expressions of several cytokines and mediators involved in inflammation [29]. To figure out whether the inhibitory effects of compounds **1** and **2** on the release of pro-inflammatory factors were through suppression the LPS-stimulated activation of NF-κB signaling, we evaluated the effects of compounds **1** and **2** on the nuclear translocation of NF-κB p65 subunit and the phosphorylation of IκB-α. In control cells, a majority of NF-κB p65 was retained in cytoplasm, while LPS treatment markedly increased p65 nuclear translocation. In the presence of compound **1**, the cytoplasm located p65 was slightly increased (Figure 6b). We also found that compounds **1** and **2** inhibited the LPS-induced phosphorylation of IκB-α in a dose-dependent manner (Figure 6c). In summary, we came to the conclusion that compounds **1** and **2** exhibited potential anti-inflammatory effects via inhibiting the MAPKs and NF-κB pathways in RAW264.7 macrophage.

## 3. Discussion

Lily bulbs have long been used as dietary supplements and traditional Chinese medicines for the treatment of chronic gastritis, pertussis, pneumonia, bronchitis and cough. The methanol extracts of lily bulbs were reported to exhibit anti-inflammatory effects in both cells and cigarette smoke-exposed mouse models [4,5]. However, no detailed research has been reported regarding the bioactive constitutes responsible for the anti-inflammatory effect of lily bulbs. In the present study, two phenylpropenoid glycerides, 1-*O*-feruloyl-2-*O*-*p*-coumaroylglycerol (**1**) and 1,3-*O*-diferuloylglycerol (**2**), were isolated from the active chloroform fraction through bioassay-guided methods and the potential anti-inflammatory activities of these two compounds were examined on LPS-induced RAW264.7 cells.

Macrophages play a critical role in acute and chronic inflammatory responses through the production of inflammatory factors, such as NO and cytokines (IL-6, TNF-α and IL-1β) [13]. Moreover, LPS-stimulated macrophages induced the high expression of iNOS to metabolize l-arginine into l-citrulline and NO [30]. Under normal conditions, NO assists the activation of macrophages through signal transduction, hence increasing the ability of macrophages to kill microorganisms. However, high levels of NO in macrophages in turn induce innate immunity and cause tissue or cell injury [31]. In addition to iNOS, COX-2, an isoform of COX, could be up-regulated by various pro-inflammatory agents, including lipopolysaccharide and cytokines. Furthermore, as a central mediator of inflammatory process, each step of cyclooxygenease-2 regulation could be a potential therapeutic target [32]. In our study, we observed that different concentrations of compounds **1** and **2** markedly inhibited the production of NO and downregulated iNOS and COX-2 protein expression in LPS-stimulated RAW 264.7 cells, suggesting that the two compounds had potential anti-inflammatory effect. A growing evidence indicates that TNF-α, IL-1β and IL-6 are critical pro-inflammatory cytokines involved in inflammation [33,34]. The over-production of TNF-α in LPS-induced RAW264.7 cells may cause tissue damage, even leading to sepsis and shock [35]. Similar to TNF-α, as another pro-inflammatory cytokine, IL-1β also induces various inflammatory diseases and IL-6 can induce C-response protein production and cause fever during the inflammatory response [36]. Therefore, TNF-α, IL-1β and IL-6 are defined as molecular targets for preventing inflammation and suppression of these pro-inflammatory cytokines secretion can delay inflammation responses and alleviate tissue injury. Our results showed that compounds **1** and **2** effectively decreased the secretion of TNF-α, IL-1β and IL-6 in messenger RNA (mRNA) and protein levels dose-dependently, suggesting they had powerful anti-inflammatory activities. In addition, we compared the anti-inflammatory effect between compounds **1**, **2** and dexamethasone. Although the pro-inflammatory cytokines inhibitory effect of compounds **1** and **2** was weaker than the positive drug dexamethasone at the same dose, they had a similar effect to dexamethasone on antagonizing the production and release of pro-inflammatory cytokines, such as TNF-α, IL-1β and IL-6. This result suggests that compounds **1** and **2** exerted anti-inflammatory effect through inhibiting the TNF-α, IL-6, and IL-1β production like dexamethasone.

Although the cellular signaling pathways regulating inflammation process are very complex, MAPKs and NF-κB pathways have been demonstrated as at least two key pathways in the modulation of inflammatory mediators including iNOS and COX-2 as well as other cytokines expressions. Additionally, it has been demonstrated that LPS activates the proteins expressions involved in MAPKs and NF-κB signaling pathways [37,38]. The MAPKs, containing JNK, ERK and p38, has been reported to modulate COX-2 and iNOS expression, and pro-inflammatory cytokines secretion in LPS-induced macrophages [28,39,40]. Our subsequent investigation showed that pretreatment with compounds **1** and **2** markedly inhibited JNK, ERK and p38 phosphorylation in LPS-activated RAW264.7 cells, indicating that the two compounds might inhibit the release of pro-inflammatory cytokines and the protein expression of iNOS and COX-2 through inhibition of the MAPK pathway. The transcription factor NF-κB, consisting of p50 and p65 subunits, also plays a significant role in the inflammatory response. In normal macrophages, NF-κB combines with its inhibitory protein (IκB) and exists in the cytoplasm as an inactive form. However, LPS-stimulated macrophages would strongly induce the phosphorylation and degradation of IκB, thus releasing NF-κB. Therefore, the NF-κB p65 subunit translocated into the nucleus to regulate the transcription of cell survival genes and induce the expression of inflammatory enzymes and cytokines. As demonstrated in our studies, compounds **1** and **2** inhibited p65 nuclear translocation and the phosphorylation of IκB-α. Thus, we speculated that the anti-inflammatory activities of the two compounds were likely due to the suppression of IκB-α phosphorylation and degradation, with a subsequent reduction of p65 nuclear translocation. Taken together, we conclude that the obvious anti-inflammatory activities of compounds **1** and **2** are probably caused by the suppression of both MAPK and NF-κB pathways.

In summary, using the bioactivity-guided isolation methods, two phenylpropenoid glycerides **1** and **2** with inhibitory effects on LPS-stimulated NO production were isolated from the bulbs of *L. brownii*. We also demonstrated that pre-treatment of RAW264.7 cells with compounds **1** and **2** can attenuate the LPS-induced production of pro-inflammatory mediators and cytokines by suppressing the activation of NF-κB and MAPKs signaling. This study not only provides evidence to support the anti-inflammatory efficacy of lily bulbs, but also sheds light on the potential use of lily as traditional Chinese medicines and functional food candidates for the prevention and treatment of inflammation related diseases.

## 4. Materials and Methods

### 4.1. Chemicals and Reagents

All chemicals were commercially available of analytical purity (Jiangsu Hanbang Science and Technology, Co., Ltd., Huai’an, China). Trypsin, streptomycin, penicillin, MTT, dimethyl sulfoxide (DMSO) and LPS were obtained from Sigma-Aldrich Chemical Co., Ltd. (St. Louis, MO, USA). Dulbecco’s modified Eagle medium (DMEM, high-glucose) was purchased from Hyclone Lab (Logan, UT, USA). Fetal bovine serum (FBS) was brought from Zhejiang Tianhang Biological Technology Co., Ltd. (Hangzhou, China). ELISA kits for TNF-α, PGE2, IL-1β and IL-6 were purchased from R&D Systems, Inc. (St. Louis, MO, USA). Anti-GAPDH, anti-PCNA (proliferating cell nuclear antigen), anti-iNOS, anti-COX-2, anti-JNK, anti-phosphorylated JNK (anti-p-JNK), anti-ERK1/2, anti-phosphorylated ERK1/2 (anti-p-ERK1/2), anti-p38, anti-phosphorylated p38 (anti-p-p38), anti-p65, anti-IκB-α, anti-phosphorylated-IκB-α (anti-p-IκB-α) mouse or rabbit antibodies were purchased from Cell Signaling Technology (Beverly, MA, USA).

### 4.2. Plant Material

The air-dried LB were harvested from Longhui County (Hunan province, China) in April 2014 and identified by Professor Mian Zhang of the Research Department of Pharmacognosy, China Pharmaceutical University. A voucher specimen (No. LB20140405) has been deposited in the Department of Natural Medicinal Chemistry, China Pharmaceutical University.

### 4.3. Preparation of the Extract, Bioassay-Guided Fractionation and Compounds Isolation

The air-dried LB (2.0 kg) were crushed into a coarse powder and ultrasonically extracted with methanol three times. Then methanol was evaporated under vacuum condition to give a residue (10.46% yield, LBM) which was suspended in water and successively partitioned with chloroform (1.43% yield, Fr. A), ethyl acetate (EtOAc) (0.93% yield, Fr. B) and *n*-butanol (2.80% yield, Fr. C). The remaining aqueous fraction was marked as Fr. D (6.12% yield). Each of these fractions was tested for its inhibitory activity against NO production in LPS-stimulated RAW264.7 macrophages. Fr. A with the strongest activity against NO release was further subjected to silica gel chromatography eluted with petroleum ether/acetone from 5:1 to 0:1. Consequently, five major fractions (Fr. A1 to Fr. A5) were obtained, among which Fr. A3 was demonstrated to possess the strongest ability to suppress NO production and was further chromatographed with medium-pressure liquid chromatography, eluted with MeOH–H_2_O (0–30 min, 40:60; 30–60 min, 60:40; 60–90 min, 80:20; 90–120 min, 100:0; *v*/*v*, 10 mL/min). The fractions were evaporated under vacuum to afford seven sub-fractions (Fr. A3-1 to Fr. A3-7), of which Fr. A3-3 was found to stand out as the most active in anti-inflammatory activity and subjected to HPLC analysis. Finally, Fr. A3-3 was purified by prep-HPLC (ACN–H_2_O, 36:64, 10 mL/min) to yield two phenylpropenoid acylglycerols **1** (10 mg) and **2** (43 mg). Data of each compound were given as below. Then, the isolated compounds were analyzed for their anti-inflammatory activities in LPS-induced RAW264.7 cells.

Compound **1**: Yellow gum. Electrospray ionization mass spectrometer (ESI-MS) (*m*/*z*): 413 [M − H]^−^; ^1^H-NMR (methanol-*d*_4_, 500 MHz) δ: 4.28 (4H, dd *J* = 2.5, 5.5 Hz, H-1,3), 4.16 (1H, *J* = 5.5 Hz, H-2), 7.66 (2H, d, *J* = 16.0 Hz, H-7′,7′′), 6.40 (1H, d, *J* = 16.0 Hz, H-8′), 6.36 (1H, d, *J* = 16.0 Hz, H-8′′), 7.44 (2H, d, *J* = 8.5 Hz, H-2′′,6′′), 6.77 (2H, d, *J* = 8.5 Hz, H-3′′,5′′), 6.80 (1H, d, *J* = 8.5 Hz, H-5′), 7.07 (1H, d, *J* = 8.0 Hz, H-6′), 7.19 (1H, s, H-2′), 3.86 (3H, s, 3′-OMe); ^13^C NMR (methanol-*d*_4_, 125 MHz) δ: 66.4 (C-1), 68.7 (C-2), 66.4 (C-3), 127.7 (C-1′), 111.9 (C-2′), 150.7 (C-3′), 149.4 (C-4′), 116.5 (C-5′), 124.2 (C-6′), 147.2 (C-7′), 115.2 (C-8′), 169.0 (C-9′), 127.2 (C-1′′), 131.2 (C-2′′), 116.8 (C-3′′), 161.3 (C-4′′), 116.8 (C-5′′), 131.2 (C-6′′), 147.0 (C-7′′), 114.9 (C-8′′), 169.0 (C-9′′), 56.5 (3′-OMe).

Compound **2**: Yellow gum. ESI-MS (*m*/*z*): 443 [M − H]^−^; ^1^H-NMR (methanol-*d*_4_, 500 MHz) δ: 4.29 (4H, d, *J* = 5.0 Hz, H-1, 3), 4.16 (1H, m, *J* = 5.0 Hz, H-2), 7.66 (2H, d, *J* = 16.0 Hz, H-7′,7′′), 6.39 (2H, d, *J* = 16.0 Hz, H-8′, 8′′), 7.18 (2H, d, *J* = 1.5, H-2′,2′′), 6.80 (2H, d, *J* = 8.5, H-5′,5′′), 7.07 (2H, d, *J* = 1.5, 8.5, H-6′,6′′), 3.86 (6H, s, 3′-OMe, 3′′-OMe); ^13^C-NMR (methanol-*d*_4_, 125 MHz) δ: 66.4 (C-1), 68.7 (C-2), 66.4 (C-3), 127.7 (C-1′), 111.9 (C-2′), 150.7 (C-3′), 149.4 (C-4′), 116.5 (C-5′), 124.2 (C-6′), 147.2 (C-7′), 115.2 (C-8′), 169.0 (C-9′), 127.7 (C-1′′), 111.9 (C-2′′), 150.7 (C-3′′), 149.4 (C-4′′), 116.5 (C-5′′), 124.2 (C-6′′), 147.2 (C-7′′), 115.2 (C-8′′), 169.0 (C-9′′), 56.5 (3′-OMe, 3′′-OMe).

### 4.4. Cell Culture

The mouse macrophage cell line RAW264.7 was obtained from Cell Bank of Shanghai Institute of Biochemistry and Cell Biology, Chinese Academy of Sciences (Shanghai, China). Cells were cultured in DMEM containing 10% (*v*/*v*) fetal bovine serum and antibiotics (100 U/mL penicillin and 100 μg/mL streptomycin) at 37 °C in a humidified atmosphere with 5% CO_2_. Cells were monitored daily and subcultured three times each week.

### 4.5. Preparation of Samples

The extract and fractions were dissolved in DMSO, each at a stock concentration of 100 mg/mL, and tested compounds were also dissolved in DMSO with concentrations of 100 mM. The final culture concentration of DMSO was less than 0.1%.

### 4.6. Measurement of Cell Viability

Cell viability was determined by MTT assay as described previously [41]. Briefly, RAW264.7 cells (5 × 10^4^ cells/mL) were cultured in 96-well plates for 12 h. Then the cells were treated with various concentrations of extract, fractions (5–100 μg/mL) and isolated compounds (3–50 μM) in the presence of LPS (1 μg/mL). After 24 h incubation, 5 mg/mL MTT solution was added to each well, and the cells were incubated for another 4 h at 37 °C. The supernatant was removed and DMSO was added to dissolve formazone crystals for 15 min. Next, the absorbance values were measured at 570 nm with a Spectra Shell Microplate Reader (Spectramax Plus 384, Molecular Devices, Sunnyvale, CA, USA). The cell viability in the control group (cells were not treated by tested samples or LPS) was set as 100%.

### 4.7. Nitrite Assay

As an indicator of NO synthesis, nitrite accumulation in the culture supernatant of LPS-stimulated RAW264.7 cells was determined using the Griess reagent [42]. RAW264.7 cells (1 × 10^6^ cells/mL) were seeded into a 96-well plate and pretreated with various concentrations of tested extracts, fractions (5–100 μg/mL) or tested compounds, followed by incubation with LPS (1 μg/mL) for 18 h. The supernatant (50 μL) was mixed with an equal volume of Griess reagent in a 96-well plate for 15 min at room temperature, and then the absorbance at 540 nm was determined using a microplate reader (Spectramax Plus 384, Molecular Devices, Sunnyvale, CA, USA). Fresh culture media were used as blanks in all experiments and N^G^-Monomethyl-l-arginine Monoacetate (l-NMMA) was used as a positive control.

### 4.8. Measurement of Pro-Inflammatory Cytokines

The levels of TNF-α, IL-1β, IL-6 and PGE2 were measured with an ELISA kits as previously described [43]. In brief, cells were cultured at the density of 1 × 10^6^ cells/mL. After incubation for 12 h, RAW264.7 cells were treated with different concentrations of tested samples and then stimulated with LPS (1 μg/mL) for 18 h. In the experiment, we used dexamethasone as the positive control. The culture supernatants were detected using a specific ELISA kit for TNF-α, IL-1β, IL-6 and PGE2 according to the manufacturer’s instructions.

### 4.9. Total RNA Extraction and qRT-PCR

The methods for total RNA extraction and qRT-PCR have been described previously [44]. Total RNA was isolated from RAW264.7 cells by using an EASYspin Plus tissue/cell RNA extraction kit (Aidlab Biotechnologies Co. Ltd., Beijing, China). The amount of RNA was quantified by measuring absorption value at 260 nm and 1 μg RNA was reverse transcribed to cDNA using the Transcriptor First Strand cDNA Synthesis Kit (Aidlab Biotechnologies Co. Ltd., Beijing, China). Thermal cycling conditions included an initial denaturation at 95 °C for 5 min, followed by 40 cycles of denaturation (10 s at 95 °C), annealing (15 s at 60 °C) and extension (15 s at 72 °C with a single fluorescence measurement), a melting curve program (60–95 °C with a 0.11 °C/s heat increase and continuous fluorescence measurement) and a cooling step to 40 °C. The Δ cycle threshold method was adopted to calculate the relative differences in mRNA abundance with a LightCycler 480 system (Roche Molecular Biochemicals, Mannheim, Germany) [45]. The data were normalized to the expression of β-actin. The results are expressed as fold changes and the qRT-PCR primers used in this study are listed in Table 3.

### 4.10. Preparation of Total and Nuclear Protein

RAW264.7 cells (1 × 10^6^ cells/mL) were incubated for 12 h in 6-well plates, and then pre-treated with different concentrations of tested compounds in the presence of LPS (1 μg/mL). After incubation for 18 h, cells were rinsed twice with ice-cold phosphate-buffered saline (PBS) and lysed on ice by using lysis buffer (C-3228, Sigma) containing protease inhibitor cocktail and phosphatase inhibitor cocktail (Sigma) for 10 min. Then, lysates were centrifuged at 12,000× *g* for 10 min at 4 °C and the cytoplasmic proteins were collected. To detect nuclear protein, cells (1 × 10^6^ cells/ mL) were extracted with NE-PER nuclear and cytoplasmic extraction reagent kits (Pierce, Rockford, IL, USA) to analyze the nuclear translocation of NF-κB subunit p65. All protein concentrations were determined by a Bradford assay kit (Bio-Rad, Hercules, CA, USA).

### 4.11. Western Blot Analysis

Equal amounts of protein samples were electrophoresed on 8%–10% sodium dodecyl sulfate polyacrylamide gel electrophoresis (SDS-PAGE) and transferred to polyvinylidene fluoride membranes (PVDF). Non-specific sites on the membrane were blocked with 5% skimmed milk in Tris-buffered saline-Tween20 (TBST) buffer for 2 h at room temperature and then the membranes were incubated with diluted respective primary antibodies against COX-2, iNOS, phosphoryled-ERK1/2, phosphoryled-p38, phosphoryled-JNK, ERK1/2, p38, JNK, GAPDH, p65, IκB-α, phosphorylated-IκB-α and PCNA overnight at 4 °C. After washing twice with TBST buffer, the membranes were incubated in horseradish peroxidase (HRP) conjugated secondary antibody solution for 2 h. Finally, bound immuno-complexes were detected using the ChemiDOC XRS+ system (Bio-Rad Laboratories, Hercules, CA, USA).

### 4.12. Statistical Analysis

All values were presented as the mean ± standard error of mean (SEM) from at least three replicate experiments. The significant difference was determined by one-way analysis of variance (ANOVA), followed by Dunnett’s post statistically hoc test where appropriate using GraphPad Prism version 5.0 (GraphPad Software, San Diego, CA, USA). *p* < 0.05 was considered statistically significant.

## 5. Conclusions

In conclusion, using bioactivity-guided isolation methods, compounds **1** and **2** isolated from air-dried LB have significant anti-inflammatory activities through inhibiting the production of NO, PGE2 and several pro-inflammatory cytokines, such as IL-1β, IL-6, and TNF-α in LPS induced RAW 264.7 macrophage cells. The molecular mechanism research indicated that the anti-inflammatory activities were mainly via the suppression of NF-κB and MAPK pathways. The results suggest that lily is a promising candidate for the prevention and treatment of inflammation.

## Figures and Tables

**Table 1:** IC_50_ values of crude MeOH extract of LB (LBM) and partitioned extracts of LBM against NO production.

Extracts/Fractions	IC_50_ (μg/mL)	Fractions	IC_50_ (μg/mL) ^a^
LBM	71.52 ± 4.52 *	Fr. A5	34.27 ± 1.34
Fr. A	32.70 ± 1.56 ^Δ^	Fr. A3-1	>50
Fr. B	64.35 ± 1.23 *	Fr. A3-2	18.30 ± 3.55
Fr. C	40.70 ± 0.92 ^Δ^	Fr. A3-3	9.32 ± 1.24
Fr. D	163.11 ± 2.31 *^,^^Δ^	Fr. A3-4	26.91 ± 1.46
Fr. A1	>50 *	Fr. A3-5	>50
Fr. A2	37.58 ± 1.25	Fr. A3-6	27.20 ± 2.72
Fr. A3	11.49 ± 0.69 *	Fr. A3-7	>50
Fr. A4	>50 *	l-NMMA ^b^	12.42 ± 2.50

^Δ^ *p* < 0.001 compared with LBM; * *p* < 0.001 compared with fraction (Fr.) A; ^a^ Mean ± SEM for *n* = 3; ^b^ Positive control. IC_50_: half maximal inhibitory concentration; LBM: crude MeOH extract of LB; NO: nitrite oxide; l-NMMA: N^G^-Monomethyl-l-arginine Monoacetate.

**Table 2:** The average percent cell viability and percent tumor necrosis factor-α (TNF-α), interleukin-1β (IL-1β) and IL-6 inhibition of the RAW264.7 cells treated with various concentrations of compounds **1** and **2** as well as dexamethasone with 1 μg/mL of LPS. Data are expressed as mean ± SD (standard deviation) of triplicates for each experiment.

Compounds	Concentration (μM)	Mean % Viability	Mean % TNF-α Inhibition	Mean % IL-1β Inhibition	Mean % IL-6 Inhibition
	10	96.9 ± 2.6	22.6 ± 1.6	28.6 ± 1.9	23.8 ± 2.1
**1**	20	94.8 ± 4.3	28.9 ± 2.7	45.3 ± 3.1	54.4 ± 2.9
	30	95.0 ± 3.7	51.3 ± 4.9	55.9 ± 3.9	78.6 ± 5.0
	10	97.4 ± 2.8	18.7 ± 1.3	12.5 ± 0.8	18.1 ± 1.2
**2**	20	95.2 ± 5.6	28.2 ± 3.1	23.6 ± 2.5	37.0 ± 3.2
	30	96.0 ± 3.8	51.1 ± 4.7	43.3 ± 2.6	85.4 ± 5.3
	10	95.8 ± 2.9	58.3 ± 4.2	62.1 ± 4.3	58.7 ± 2.3
**Dexamethasone**	20	101.2 ± 3.6	72.4 ± 3.2	69.7 ± 5.8	82.9 ± 4.7
	30	97.2 ± 5.8	86.4 ± 6.2	80.4 ± 7.3	91.5 ± 7.9

**Table 3:** Primers used for the qRT-PCR study.

Gene	Sequence (5′ to 3′)
iNOS	Fw:GAATCTTGGAGCGAGTTGTGGA
	Rv:GTGAGGGCTTGGCTGAGTGAG
COX-2	Fw:CTGGTGCCTGGTCTGATGATGT
	Rv:AGTCTGCTGGTTTGGAATAGTTGCT
TNF-α	Fw:CTTGTTGCCTCCTCTTTTGCTTA
	Rv:CTTTATTTCTCTCAATGACCCGTAG
IL-1β	Fw:TGTGTTTTCCTCCTTGCCTCTGAT
	Rv:TGCTGCCTAATGTCCCCTTGAAT
IL-6	Fw:AAGGAGTGGCTAAGGACCAAGAC
	Rv:AGTGAGGAATGTCCACAAACTGATA
β-actin	Fw:ACCACACCTTCTACAATGAG
	Rv:ACGACCAGAGGCATACAG
